# Quantifying the evolution of atomic interaction of a complex surface with a functionalized atomic force microscopy tip

**DOI:** 10.1038/s41598-020-71077-9

**Published:** 2020-08-24

**Authors:** Alexander Liebig, Prokop Hapala, Alfred J. Weymouth, Franz J. Giessibl

**Affiliations:** 1 Institute of Experimental and Applied Physics, University of Regensburg, 93040 Regensburg, Germany; 2 Department of Applied Physics, Aalto University, Aalto, Finland; 3 Institute of Physics, Czech Academy of Sciences, Cukrovarnická 10, 162 00 Prague 6, Czech Republic

**Keywords:** Materials science, Nanoscience and technology, Physics

## Abstract

Terminating the tip of an atomic force microscope with a CO molecule allows data to be acquired with a well-known and inert apex. Previous studies have shown conflicting results regarding the electrostatic interaction, indicating in some cases that the negative charge at the apex of the CO dominates, whereas in other cases the positive charge at the end of the metal tip dominates. To clarify this, we investigated $\hbox {CaF}_{2}$(111). $\hbox {CaF}_{2}$ is an ionic crystal and the (111) surface does not possess charge inversion symmetry. Far from the surface, the interaction is dominated by electrostatics via the negative charge at the apex. Closer to the surface, Pauli repulsion and CO bending dominate, which leads to an unexpected appearance of the complex 3-atom unit cell. We compare simulated data in which the electrostatics are modeled by point particles versus a charge density calculated by DFT. We also compare modeling Pauli repulsion via individual Lennard–Jones potentials versus a total charge density overlap. In doing so, we determine forcefield parameters useful for future investigations of biochemical processes.

## Introduction

Over the past decade, atomic force microscopy (AFM)^1^, in particular non-contact AFM (nc-AFM) at cryogenic temperatures, has evolved into a microscopy technique with unprecedented spatial resolution. A major breakthrough in nc-AFM was the ability to resolve the internal structure of simple organic molecules in real space^2^, which was enabled by functionalizing a metallic AFM tip apex with a carbon-monoxide (CO) molecule. Since then, CO-terminated tips (CO tips) have been widely applied in AFM experiments to study molecular adsorbates^3–9^ and various types of surfaces and adsorbates with atomic resolution^10–15^. The interaction of a CO tip with a sample surface is composed of different physical mechanisms, including van der Waals attraction and Pauli repulsion, which can be described by a Lennard–Jones potential^12,16,17^, and electrostatic interaction between the complex electric field of the CO tip and the sample electron density^12,13,18^. Recently, an additional transition from a physisorbed to a chemisorbed interaction state has been discovered in the interaction of CO tips with single iron adatoms adsorbed on a copper surface^19^. While the tip-sample interaction is dominated by different mechanisms at different tip-sample distances, interpretation of AFM images obtained with CO tips is additionally complicated by the lateral deflection of the CO at the tip apex, if lateral forces act between tip and sample, which can lead to image distortions and an elongated appearance of atomic-scale features in AFM images^5,16,17,20–24^.

AFM is capable of resolving the lattice of insulating substrates with atomic resolution^25,26^. Previously, Ellner et al. considered AFM images of Cl vacancies in NaCl thin films with a CO tip and showed that for such a charged feature the interaction with the strong background metal tip dipole dominates the electrostatic tip-sample interaction^12^, similarly to the case when imaging the atomic lattice of hexagonal boron nitride^13^. On the contrary, the strongly spatially localized negative charge density at the tip apex is relevant when imaging the flat NaCl lattice^12,27^. These findings raise the question as to whether the atomic-scale electrostatic AFM contrast measured with CO tips on bulk insulators can be generally explained with a negative tip apex.

**Figure 1:**
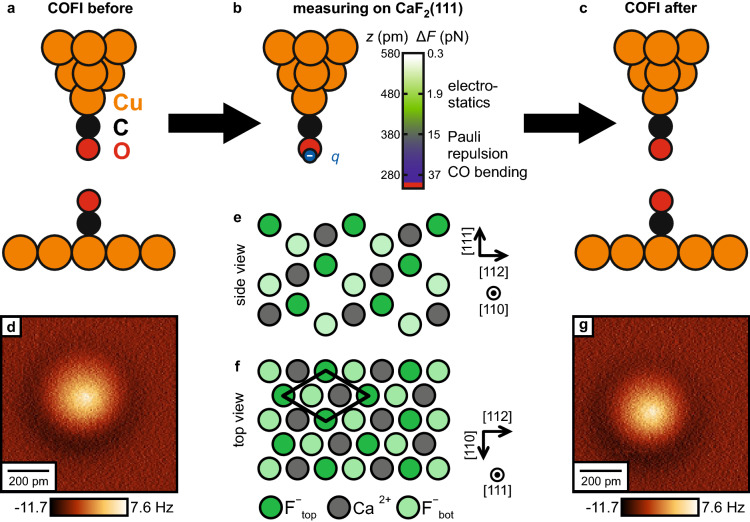
(**a**–**c**) Illustration of the measurement cycle in the experiment. After preparing and characterizing the tip on Cu(111) (**a**), the tip is transferred to a $\hbox {CaF}_{2}$(111) sample for the measurements (**b**), and then again back to a Cu(111) sample to verify that the tip did not change during the whole experiment (**c**). The COFI images (**d**,**g**) recorded before and after the measurement on $\hbox {CaF}_{2}$(111) show that the tip remained stable for the complete measurements. Imaging height: 70 pm retracted from the STM setpoint height on the bare Cu(111) surface, (${-10}\hbox { mV}$, $-100\hbox { pA}$). (**b**) A CO-terminated tip has been modeled with a negative point charge *q*. Graph: Plot of the calculated force contrast $\Delta F$ and responsible tip-sample interactions as a function of tip-sample distance *z* above $\hbox {CaF}_{2}$(111). (**e**,**f**) Side and top view of the $\hbox {CaF}_{2}$(111) surface, consisting of neutral F$^-$–Ca$^{2+}$–F$^-$ triple layers^32,39^.

In this work, we investigate the imaging mechanisms of CO tips on the ionic $\hbox {CaF}_{2}$(111) surface. Previous studies of the CO tip imaging mechanisms on ionic lattices focused on atomically-flat NaCl films^12^. Fourfold-symmetric crystal surfaces of the rock salt structure possess charge inversion symmetry, as multiplying the ionic charges with $-1$ just shifts the atomic pattern by half a cubic lattice vector. As a consequence, in previous AFM studies atomic identification of sample atoms required indirect theoretical characterization^28,29^, or adsorbed marker molecules on the surface^30,31^. In contrast, the threefold-symmetric $\hbox {CaF}_{2}$(111) surface lacks charge inversion symmetry and the surface atomic layer consists solely of F$^-$ ions, with the second layer of Ca$^{2+}$ ions ${79}\,\hbox {pm}$ below the surface layer (Fig. 1e,f)^32,33^. For this reason, the surface has been of particular interest to AFM studies to identify the tip apex polarity^34–38^. The complex interplay of ionic charges and different atomic heights makes the $\hbox {CaF}_{2}$(111) surface an ideal model surface to study the relative contributions of electrostatic interaction, van der Waals attraction and Pauli repulsion between an ionic surface and a CO tip, and to characterize the effect of CO bending on the AFM contrast on the basis of a corrugated surface.

We acquired AFM data of the $\hbox {CaF}_{2}$(111) surface covering a tip-sample distance range of $300\hbox { pm}$. Upon approach, the atomic contrast is initially governed by short-range electrostatic interaction between the negative charge density in front of the O at the tip apex and the electric field of the sample surface and increases exponentially with decreasing tip-sample distance. The atomic scale AFM contrast can be reproduced by an electrostatic calculation, where the tip is represented by a single negative point charge^27^, because Pauli repulsion and van der Waals attraction partly compensate each other and decay faster than short-range electrostatics, leaving the latter as the dominant contribution. If the tip-sample distance is further reduced, short-range Pauli repulsion starts to dominate the AFM contrast. In this regime, strong lateral forces cause lateral deflection of the CO, which leads to a strong contrast variation in AFM images at smallest tip-sample distances, that can be reliably explained using the probe particle model^17^. By a combination of experiment and theory we are able to characterize the complete contrast formation on the corrugated $\hbox {CaF}_{2}$(111) surface with CO-terminated tips.

## Results

Figure 1a–c illustrates the measurement cycle: after the tip is successfully terminated with a CO molecule on the Cu(111) surface, the tip is characterized by scanning over a second CO molecule. This CO Front Atom Identification method (COFI; see “Methods” for details)^11,40,41^ allows us to verify that the tip has not changed during the measurement cycle. Then, the Cu(111) sample is removed and replaced with the $\hbox {CaF}_{2}$(111) sample for data acquisition. After all $\hbox {CaF}_{2}$ data has been acquired, the $\hbox {CaF}_{2}$ sample is removed and replaced again by the Cu(111) sample so that we can perform a second COFI characterization. The COFI images of the CO tip recorded before and after the measurement are shown in Fig. 1d,g, respectively. At small tip-sample distances, the CO-CO interaction results in a bright, circularly-symmetric feature in constant-height AFM images^3,42^. The two COFI images are equal, which confirms that the atomic composition of the tip apex did not change during the measurement on $\hbox {CaF}_{2}$(111) (see Supplementary Fig. S1 for additional analysis).

**Figure 2:**
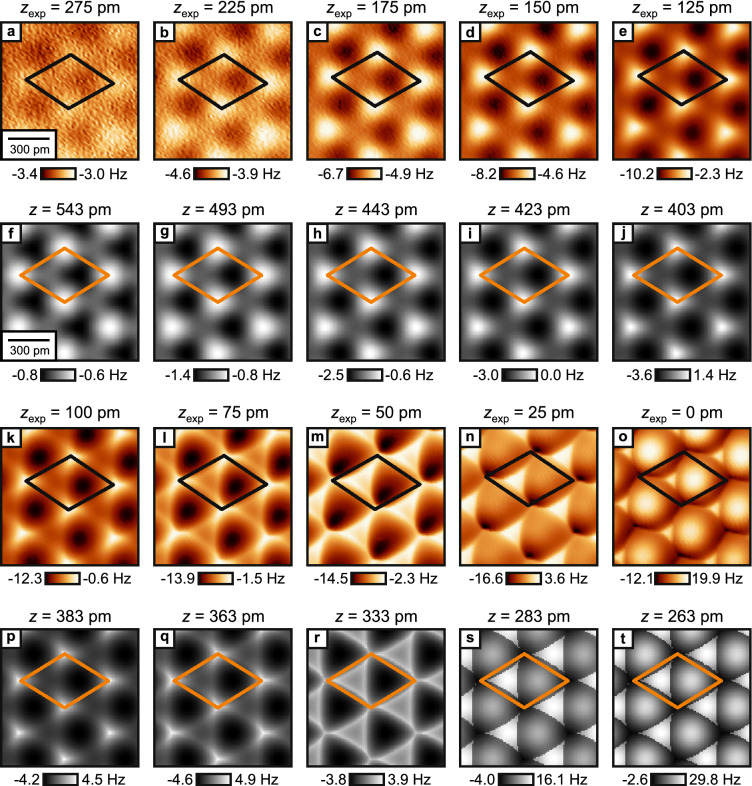
(**a**–**e**, **k**–**o**) Experimental constant-height $\Delta f$ images as a function of tip-sample distance (raw data) measured with a CO tip on $\hbox {CaF}_{2}$(111) at the same spot on the sample. $z_{\text{exp}} = 0\,\hbox {pm}$ is defined as the closest tip-sample approach in the experiment. (**f**–**j**, **p**–**t**) Constant-height images calculated with the probe particle model for a CO tip on $\hbox {CaF}_{2}$(111) over a similar tip-sample distance range as the experimental images. The calculation reproduces all measured contrast in good agreement. *z* is defined as the vertical distance between the F$^-_{\text{top}}$ and the tip apex oxygen nuclei. The unit cell defined in Fig. 1f is drawn in all images.

Figure 2a–e,k–o shows experimental constant-height images recorded of the same spot on the surface as a function of tip-sample distance. Initially, the images present three high-symmetry sites: a local minimum (dark), a local maximum (bright) and a saddle point (intermediate) and the contrast increases upon approach (Fig. 2a–d). From symmetry considerations it follows that these high-symmetry sites correspond to the atoms of the surface triple layer of $\hbox {CaF}_{2}$(111) (see Fig. 1e,f). When the tip-sample distance is further reduced (Fig. 2e,k,l), a sharpening of features can be observed, which has previously been attributed to CO bending in images recorded of organic molecules^16,43^. By further decreasing the tip-sample distance, the contrast between the three sites starts to change drastically (Fig. 2m,n), until the surface appears as a hexagonal arrangement of bright spheres in the image at closest approach (Fig. 2o). The distance $z_{\text{exp}} = 0\,\hbox {pm}$ is defined as the closest tip-sample approach in the experiment.

To understand the rich contrast features observed in the experimental constant-height images, we performed AFM image simulations with a modified version of the probe particle model^17^. The contribution of Pauli repulsion, obtained by calculating the overlap of the electron densities of tip and sample, is added to the attractive van der Waals component^18^. Additionally, Coulomb interaction is added via a convolution between the electrostatic sample potential and the tip’s electron density that were obtained independently from density functional theory calculations (see “Methods” for further details). Figure 2f–j,p–t shows calculated constant-height $\Delta f$ images, obtained with the probe particle model as a function of tip-sample distance over a similar range as the experimental images shown in Fig. 2a–e,k–o. The tip-sample distance *z* is defined as the vertical distance between the F$^-_{\text{top}}$ and the tip apex oxygen nuclei. While the experimental images show continuous transitions with reducing the distance from the deep non-contact regime to close contact where significant bending occurs, we have not followed the strict $50\hbox { pm}$ and $25\hbox { pm}$ distance decrements for the selection of the calculated images. Instead, we chose distances that provided an optimized match to the experimental images (e.g. $\Delta z_{\text{exp}} = 25\hbox { pm}$ between Fig. 2d and e compared to $\Delta z = 20\hbox { pm}$ between Fig. 2i and j). The model reproduces the experimentally observed contrast in good agreement both qualitatively as well as quantitatively. A slight difference is the shrinking of the bright triangular features in the experimental images at closest tip-sample distances (left halves of the unit cells in Fig. 2m–o), which is not visible in the simulated images (Fig. 2r–t). This shrinking is visible in the simulation only at slightly closer tip-sample distances (see Supplementary Fig. S2 for additional simulated images). Note that overall the $\Delta f$ values obtained from the probe particle model are offset to more positive values as compared to the experimental values. This can be attributed to the attractive offset that is added in the experiment due to the long-range van der Waals interaction which is not included in the probe particle model.

**Figure 3:**
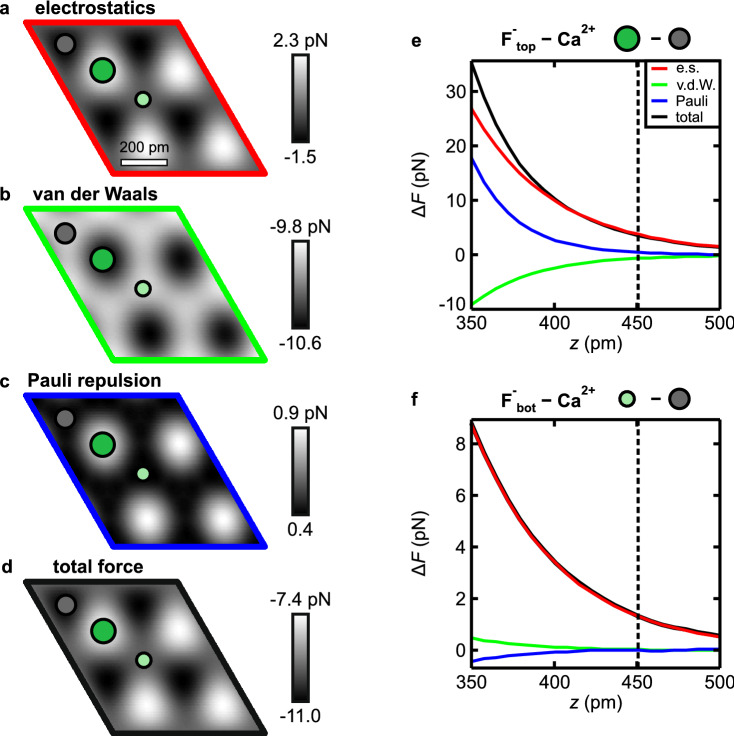
(**a**–**c**) Interaction decomposed images [of a $\hbox {CaF}_{2}$(111) supercell] at a tip-sample distance of $z = 451\,\hbox {pm}$, and (**d**) the total force obtained by summing all three contributions obtained with the probe particle model. (**e**,**f**) Individual contributions to the atomic force contrasts $\Delta F$ as a function of tip-sample distance *z*. The dashed line marks the tip-sample distance of the images in (**a**–**d**). The colors match the frames of (**a**–**d**).

Initially (Fig. 2f–i), we assert that, in agreement with Ref. ^12^, the contrast is mainly formed by short-range electrostatic interaction between tip and surface, and increases upon approach. Above the atoms of the surface F$^-$-layer, the electrostatic repulsion between the ions and the negative charge density at the CO tip apex leads to a decrease of the overall attractive tip-sample interaction, whereas above the Ca$^{2+}$ atoms, the electrostatic attraction increases the attractive tip-sample interaction. We have decomposed the individual contributions to the overall tip-sample force from the probe particle model (Fig. 3a–c). The total force (Fig. 3d) is obtained by summing the three individual contributions and closely resembles the pattern that is obtained only by short-range electrostatics (Fig. 3a).

The strong height differences between the atoms in the surface triple layer are key to understanding why the AFM contrast in this regime is explained solely by short-range electrostatics: The individual contributions of van der Waals attraction, Pauli repulsion and electrostatic interaction as a function of tip-sample distance are shown in Fig. 3e,f. The force contributions from atomic van der Waals attraction (green) and Pauli repulsion (blue) decay faster than the electrostatic interaction (red). Therefore in this tip-sample distance regime, a sizable contribution to the overall interaction due to these two components is obtained only above the atoms in the surface F$^-$-layer, that are approximately $79\hbox { pm}$ closer to the tip than the Ca$^{2+}$ atoms in the second layer (see Fig. 3b,c). Additionally, the opposite nature of the two forces leads to a cancellation effect: the attractive van der Waals attraction is partly compensated by Pauli repulsion, which contributes to the fact that the electrostatic interaction is the dominant contribution to the atomic scale AFM contrast at large tip-sample separations. Note that this calculation of the interaction was performed for a rigid probe particle, however, at this tip-sample distance regime CO bending has only a negligible influence on the measured contrast.

**Figure 4:**
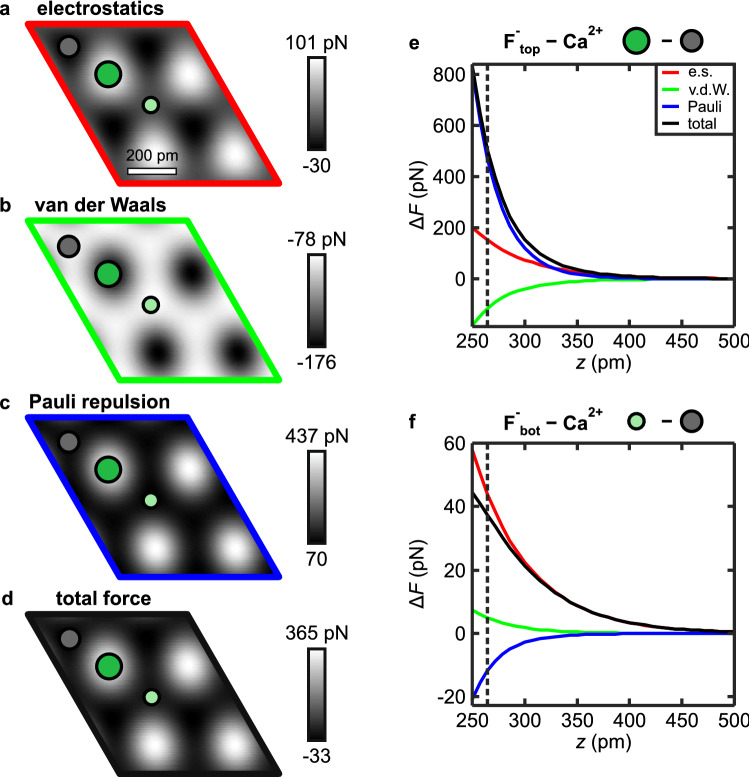
(**a**–**c**) Interaction decomposed images [of a $\hbox {CaF}_{2}$(111) supercell] at a tip-sample distance of $z = 272\hbox { pm}$, and (**d**) the total force obtained by summing all three contributions obtained with the probe particle model. (**e**,**f**) Individual contributions to the atomic force contrasts $\Delta F$ as a function of tip-sample distance *z*. The dashed line marks the tip-sample distance of the images in (**a**–**d**). The colors match the frames of (**a**–**d**).

If the tip-sample distance is decreased, the probe particle starts to deflect laterally due to an increase of the lateral component in the tip-sample force, which results in a sharpening of the atomic features in the constant height images (Fig. 2j,p,q), as also seen in the experimental results (Fig. 2e,k,l). Upon further approach, the probe particle model (Fig. 2r–t) correctly reproduces the experimentally observed contrast patterns (Fig. 2m–o). To determine the dominant interaction at this tip-sample distance regime, we again decomposed the total force (Fig. 4a–d), revealing that Pauli repulsion is the dominant contribution to the total AFM contrast with strong repulsive features above the surface F$^-_{\text{top}}$ ions (Fig. 4c). This is further illustrated by the individual force contrast versus distance plots in Fig. 4e,f, showing that the contrast caused by Pauli repulsion above the F$^-_{\text{top}}$ ions (blue curve in Fig. 4e) is largest in magnitude. Having addressed the relevant physics at this tip-sample distance regime we can discuss the influence of CO bending on the AFM contrast. In the image recorded at the smallest tip-sample separation (Fig. 2o), the AFM contrast pattern resembles the hexagonal arrangement of bright spheres. This pattern would not be obtained by simply adding the three interactions obtained for a rigid probe particle (Fig. 4a–c), which would yield a strongly repulsive feature above the surface F$^-$ atoms (Fig. 4d). If the probe particle is allowed to relax, it will slide around the exposed atoms of the surface F$^-$ layer, which completely alters the observed AFM contrast. This mechanism leads to the above-mentioned appearance of bright, repulsive sphere-like features at the positions of the Ca$^{2+}$ atoms and triangular features at the positions of the F$^-_{\text{bot}}$ atoms in the AFM images. Above the surface F$^-_{\text{top}}$ atoms, CO bending causes the occurrence of sharp attractive ridges (see Fig. 2o,t, the corners of the unit cells are at the F$^-_{\text{top}}$ positions).

Based on the above analysis we can now discuss the AFM contrast formation of CO tips on $\hbox {CaF}_{2}$(111) as a function of tip-sample distance. At large tip-sample distances, short-range electrostatics dominate the AFM contrast, while Pauli repulsion and van der Waals attraction compensate each other. If the tip-sample distance is decreased, Pauli repulsion starts to overcome the electrostatic interactions at a tip-sample distance of about $z = 300\hbox { pm}$, while the strongest repulsion is observed above the protruding F$^-_{\text{top}}$ atoms. At this point, CO bending has additional influence on the images, as this leads first to a sharpening of features and then, at even closer distances, to contrast inversion^43^. The observation of a transition from an electrostatic imaging regime to a regime where Pauli repulsion dominates the AFM contrast is in agreement with the findings of Ellner et al.^12^. This suggests that the interaction of a CO tip with an ionic crystal is generally dominated by short-range electrostatics at larger tip-sample distances, where the negative charge density at the tip apex is responsible for the atomic contrast, and Pauli repulsion at close tip-sample distances.

**Figure 5:**
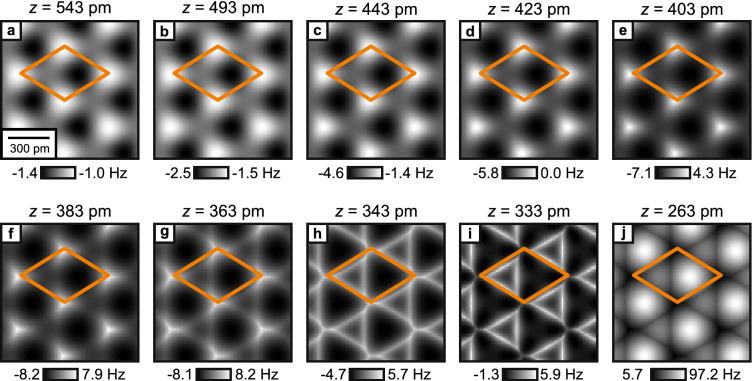
Calculated *z*-dependent constant-height images created with the probe particle model using the standard choice of Lennard–Jones potentials to describe van der Waals attraction and Pauli repulsion. The unit cell defined in Fig. 1f is drawn in all images.

In the above calculation, we used a modified version of the probe particle model, calculating the overlap of electron densities to obtain the contribution of Pauli repulsion. For comparison, we have also performed calculations using the standard method of the model that is based on Lennard–Jones potentials as formulated in Ref. ^17^ to describe van der Waals attraction and Pauli repulsion. Electrostatic interaction is added as a convolution of the sample electrostatic potential and a quadrupole-type charge distribution modeling the CO tip (see “Methods”). Figure 5 shows constant-height images of the $\hbox {CaF}_{2}$(111) surface calculated with this standard approach over a similar *z* range as the experimental and calculated images in Fig. 2. Both simulations give similar results that reproduce the experimental images especially at larger tip-sample distances (Fig. 5a–g). Strong discrepancies between both simulations occur only at smallest tip-sample distances: In the density overlap simulation (Fig. 2r,s) the experimentally observed appearance of a bright triangular feature in the left half of the unit cell (Fig. 2m,n) is nicely reproduced. In contrast, in the images created from the simulation using Lennard–Jones potentials (Fig. 5h,i) these features appear with a dark center and bright ridges instead of a triangular feature with a bright center. On the contrary, the shrinking of this bright triangle observed in the experimental data at closest tip-sample distance (Fig. 2o) is better reproduced by the standard probe particle model approach (Fig. 5j) as compared to the density overlap simulation (Fig. 2t), where this shrinking is observed at slightly smaller tip-sample separations (Supplementary Fig. S2). Note that overall the $\Delta f$ contrast is slightly overestimated in the simulation incorporating Lennard–Jones potentials as compared to the experimental contrast. For a direct comparison of both simulations, we show all calculated images from Figs. 2 and 5 as a single figure in the Supplementary information (Supplementary Fig. S3).

Having addressed all physical mechanisms relevant in the contrast formation, we can investigate the electrostatic imaging regime in more detail. As described above, the electrostatic tip-sample interaction is included in the probe particle model by a convolution of the electrostatic potential of the sample and the tip’s electron density. Previous works incorporating electrostatic tip-sample interactions used a single negative point charge to represent the CO tip apex^24,27^. To quantify the success of such a simple point charge model, we compare our data to a calculation where the sample atoms are represented by point charges and the CO tip apex by a single negative point charge $q = -0.03\,e$, where *e* denotes the elementary charge (see “Methods”)^39^.

**Figure 6:**
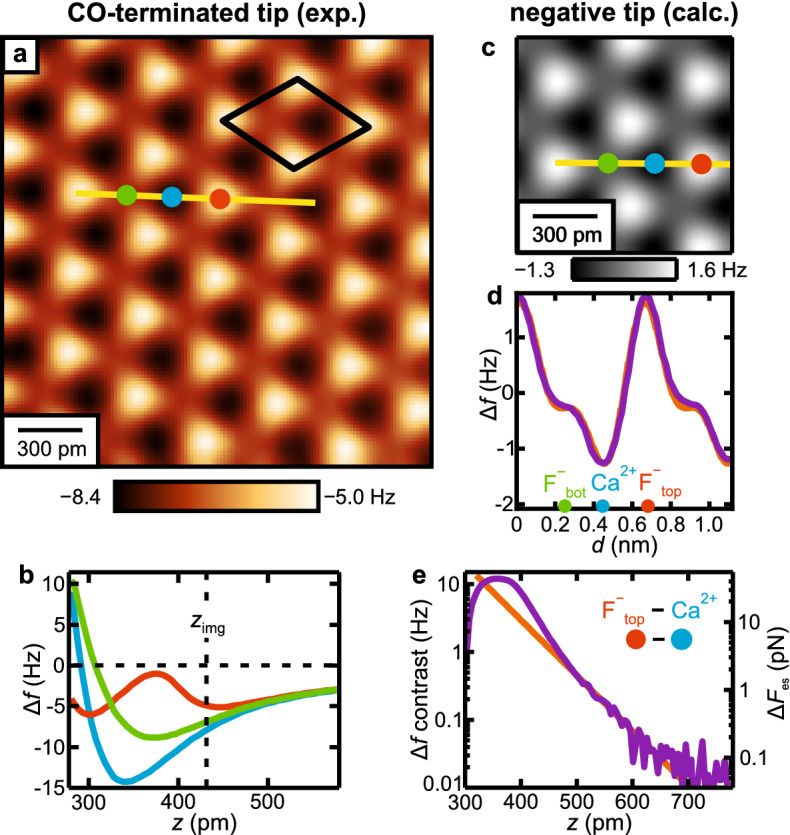
(**a**) Experimental constant-height $\Delta f$ image of the $\hbox {CaF}_{2}$(111) surface recorded with a CO-functionalized tip, processed with a 78 pm $\times$ 78 pm Gaussian low-pass filter^44^. The unit cell defined in Fig. 1f is drawn black in the image. (**b**) Experimental $\Delta f(z)$ curves recorded above the three high-symmetry sites marked in (**a**). The constant-height image [(**a**)] has been recorded at the tip-sample separation $z_{\text{img}}$. (**c**) Calculated constant-height $\Delta f$ image for a negatively-terminated tip using the electrostatic point charge calculation. (**d**) Comparison of experimental (purple) and and calculated (orange) line profiles following the traces in the respective images [(**a**) and (**c**)]. To align the experimental to the calculated data, the average over one period of the profile has been subtracted from each curve. Note that the overall $\Delta f$ contrast in (**a**) is slightly higher than in the line profile shown in (**d**). This can be attributed to the fact that the imaging plane is not perfectly aligned with the sample, leading to a slightly darker contrast in the top left corner as compared to the brighter bottom right corner. (**e**) Comparison of experimental and calculated $\Delta f(z)$ contrasts, together with the calculated electrostatic force contrast $\Delta F_{\text{es}}$.

Figure 6a shows a constant-height frequency shift $\Delta f$ image of the $\hbox {CaF}_{2}$(111) surface recorded with the CO tip at a tip-sample distance of $z = 430\hbox { pm}$, i.e. at a tip-sample distance regime where short-range electrostatics dominate the AFM contrast, as verified with the probe particle model (Fig. 3d,e). Frequency shift versus distance $\Delta f(z)$ spectra recorded on the three sites are shown in Fig. 6b. The constant-height image in Fig. 6a has been recorded at $z_{\text{img}}$, indicating that the overall tip-surface interaction is attractive in this *z*-range. Figure 6c shows a calculated constant-height $\Delta f$ image for the CO tip obtained from the electrostatic calculation. Similar to the experimental constant-height image and the probe particle model (Fig. 2f–i), the image from the electrostatic calculation presents three prominent sites that correspond to the atoms in the top $\hbox {CaF}_{2}$(111) triple layer: the Ca$^{2+}$ atoms are imaged dark, i.e. most attractive, the atoms of the surface F$^-$-layer are imaged bright, i.e. least attractive, and the atoms of the lower F$^-$-layer correspond to the sites of intermediate contrast.

Figure 6d shows an experimental (purple) and a calculated line profile (orange), extracted along the high-symmetry directions in Fig. 6a,c, respectively. As shown in Ref. ^39^, we calculate the agreement between experiment and calculation as $$1- {\text{RQD}}$$, where RQD is the relative quadratic deviation over one surface period in the line profiles. For the data shown in Fig. 1, we obtain an agreement of 99.6 %, which illustrates the success of the simple point charge model to reproduce the electrostatic interaction of CO tips with ionic lattices. This finding becomes even more evident when the $$\Delta f$$ contrast is plotted as a function of tip-sample distance *z*^45^. As shown previously, the *z*-component of the electric field decays exponentially above an ionic lattice, where the decay length $$\lambda = 1 / a^*$$ is given by the length of the surface reciprocal primitive lattice vector $$a^*$$^39,46^. For the $$\hbox {CaF}_{2}$$(111) surface, this leads to a decay length of $$\lambda = 53.2\hbox { pm}$$^39^. Hence, the force acting on a point charge in the electric field of the surface and the resulting $$\Delta f$$ will show the same dependence on *z*, as clearly visible for the calculated curve (orange) in Fig. 6e. The experimental $$\Delta f$$ contrast (purple) also shows an exponential decay for *z*-values larger than $$450\hbox { pm}$$, with a decay length of $$\lambda _{\text{exp}} = (53\pm 3)\,{\text{pm}}$$, which matches the expected decay rate of the electric field. At closer tip-sample distances ($$z < 450\hbox { pm}$$) the experimental spectrum deviates from the exponential behavior, and the electrostatic calculation can no longer reproduce the measured contrast.

## Discussion

When comparing the images obtained with a single-atom metal tip in Ref. ^39^ to the images obtained with the CO tip presented here, we find in agreement to previous studies an inversion of the AFM contrast in the electrostatic imaging regime^12,27^. This contrast inversion is a result of the opposite effective tip apex polarities when imaging an ionic lattice. Metal tips present a positive pole at the tip apex due to the Smoluchovski effect^27,47–49^, while the negative charge density in front of the O atom at the CO tip apex is relevant when imaging an ionic lattice^12,13^. At the largest tip-sample distance at which we observe atomic resolution, we showed that a single, negative point charge is sufficient to model the short-range electrostatic interaction of the CO tip with a defect-free ionic lattice. Upon approaching the surface, the atomic contrast is governed by Pauli repulsion and the CO at the tip apex is subject to strong lateral deflections around the exposed surface F$^-$ atoms, leading to a complete change of the AFM contrast. The mechanical probe particle model successfully reproduced the unique contrast patterns in this regime, which illustrates the validity of this widely applied model to reproduce the CO bending mechanism even for a corrugated, ionic crystal surface. Based on this analysis, we suggest imaging ionic crystals with atomically-characterized tips that possess a known tip apex polarity in an electrostatic interaction regime for the determination of chemical species based on AFM data.

We applied two different probe particle model calculations yielding similar results. Therefore, this comparison can be used to compare the degree of correspondence between a Lennard–Jones based approach and the density overlap method by Ellner et al.^18^. The ability of switching between two different methods and still obtaining similar results nicely demonstrates the versatility of the probe particle code. Additionally, in common classical forcefields there are no good estimates of Lennard–Jones radii valid for ionic crystal surfaces. For this reason, the ionic radius needs to be modified in models based on Lennard–Jones potentials, as previously done in the case of sodium cations hydrated by water on a NaCl surface^8^. In the calculation that relies on the overlap of electron densities^18^, the ionic radius is obtained *ab initio*. Therefore, our approach of combining high-resolution AFM imaging with detailed simulations can be used for the validation of ionic radii used in classical forcefields, which could be interesting for the development of biochemical simulations of processes like the interaction of proteins or DNA with ions on surfaces and in solution that can be done with molecular forcefield simulations like AMBER^50^. We would like to note that the probe particle model method, where the electric field of the CO tip is modeled as a quadrupole charge distribution, has been previously employed by Schulz et al. in Ref. ^13^ to simulate images of a Cl vacancy in a NaCl lattice. The calculations in Ref. ^13^ are in good agreement to the work of Ellner et al. on the same sample system^12^, which shows that this approach is not only valid for an atomically-corrugated $\hbox {CaF}_{2}$(111) surface, but also for a flat NaCl lattice.

In combination with its capability of sensing a single electric charge^51–57^, AFM has recently opened the way for studies of previously inaccessible nanoscale processes, like the measurement of charge transfer between two molecules^58^, determination of the reorganization energy upon charging^59^, and imaging of molecular orbitals with sub-molecular resolution on a bulk insulator surface^60,61^. Lately, the high-resolution imaging capabilities of AFM using CO tips have been combined with the ability to control the charge state of a single molecule on an insulating substrate^62^. In this context, the determination of adsorption geometries of molecules on insulator surfaces by means of AFM utilizing CO tips requires a thorough characterization of the atomic-scale imaging mechanisms of CO tips on bulk insulators. Especially the $\hbox {CaF}_{2}$(111) surface, which can be reliably grown on Si(111)^63^, has been shown to be a promising substrate candidate for room-temperature applications of molecular anchoring^64–66^.

We have reported AFM experiments on the complex ionic $\hbox {CaF}_{2}$(111) surface with CO-terminated metal tips, combined with theoretical modeling of the AFM contrast using a state-of-the-art model to reproduce AFM images with functionalized tips^17^. In this way, we were able to characterize all relevant imaging mechanisms when probing a corrugated ionic lattice with CO tips, and obtained qualitative and quantitative agreement between experiment and theory. In conclusion, the precise understanding of the atomic contrast measured with CO tips opens the possibility of atomically-precise determination of molecular adsorption positions combined with submolecular resolution imaging on the $\hbox {CaF}_{2}$(111) surface.

## Methods

### Experimental details

The experiments have been conducted with a commercial low temperature combined scanning tunneling/atomic force microscope (LT STM/AFM, Scienta Omicron GmbH, Taunusstein) in ultrahigh vacuum (UHV) at a temperature of $4.4\hbox { K}$. We used a qPlus sensor (type qPlus M4)^67^ equipped with an iridium tip, that was sharpened with a focused-ion-beam (FIB), showing a resonance frequency of $f_0 = 55051\hbox { Hz}$, a stiffness of $k = 1800\hbox { Nm}^{-1}$, and a quality factor of $Q = 811485$. The sensor was operated in the frequency modulation mode (FM-AFM)^68^ at a constant amplitude of $A = 50\hbox { pm}$. In FM-AFM the frequency shift $\Delta f$ of the sensor from its unperturbed resonance frequency $f_0$ is a measure of the vertical tip-sample force gradient. In the measurements on $\hbox {CaF}_{2}$(111), the sample bias $V_{\text{b}}$ was set to minimize the long-range electrostatic tip-sample interaction. We therefore recorded $\Delta f(V_{\text{b}})$ curves, so called Kelvin parabolas and used the apex voltage $V_{\text{CPD}}$ as the imaging voltage (see Supplementary Fig. S4). The position of the apex in the Kelvin parabola is slightly different for spectra measured above the three high-symmetry sites of ${\hbox {CaF}}_{2}$(111) and we determined the actual imaging voltage $V_{\text{b}} = + 22.5\,\, {\text{V}}$ by averaging the apex positions in all three spectra.

We prepared our tips on a Cu(111) sample that was prepared using standard sputter-anneal cycles. For tip functionalization, gaseous CO was leaked into the chamber onto the cold sample surface until a coverage of about $0.01\hbox { ML}$ CO was obtained. The $\hbox {CaF}_{2}$(111) sample was cleaved in ambient conditions and then transferred to the UHV system. To remove contaminants from the surface, it was annealed several hours at about $550\,^{\circ }\mathrm{C}$.

Before we functionalized the tip apex with CO, we prepared metal tips ending in a single Cu atom by repeated indentations between $300\hbox { pm}$ and $1\hbox { nm}$ into the Cu(111) surface^69^. Afterwards, we characterized the tip with the Carbon-Monoxide-Front-Atom-Identification (COFI) method^11,40,41^, and repeated the process of tip poking and COFI characterization until we obtained the COFI portrait of a single-atom metal tip. In COFI, the tip is scanned at a constant height above a CO molecule adsorbed on a copper surface. Since the CO adsorbs upright on Cu(111) with the O side pointing away from the surface, the CO acts as a probe and the first atomic layer of the tip apex can be resolved in the $\Delta f$ images. Afterwards, CO functionalized tips were prepared following the procedure described by Bartels et al.^70^. After the tips were successfully functionalized with carbon monoxide, the samples were exchanged and the tip was approached to the $\hbox {CaF}_{2}$(111) sample. During this procedure, the tip was kept cold to prevent any thermally induced tip changes. After data was acquired on $\hbox {CaF}_{2}$(111), changes in the atomic structure of the tip apex during the measurements were excluded by investigating the tips again on Cu(111) with the COFI method. A data set was considered in the analysis only if the COFI portraits recorded before and after the measurement on $\hbox {CaF}_{2}$(111) were equal (see Fig. 1a–c for an illustration of the measurement cycle).

### AFM image simulations using the probe particle model

We conducted two AFM simulations with the probe particle model using two different methods for the approximation of Pauli repulsion. The **standard method** is based on Lennard–Jones potentials as formulated in Ref. ^17^ with the electrostatic force calculated by a convolution of the DFT-calculated electrostatic potential of the sample with a quadrupole charge distribution ($dz^2$-orbital) to model the tip (see SI of Refs. ^9^ and ^71^), normalized to a quadrupole of $-0.1\,e{\AA }^2$. The Lennard–Jones radius of the calcium cation was modified with respect to default parameters to 1.70 Å. The similarly determined radius of the fluorine anion was consistent with the default value of 1.75 Å. These ionic radii were determined by analysis of the electron density from DFT calculations taking an isosurface value of $0.01\hbox { eV}/{\AA }^{3}$. Note that we previously used a similar method for the estimation of the ionic radius of a sodium cation hydrated by water on a NaCl surface^8^, since in common classical forcefields there are no good estimates of Lennard–Jones radii valid for ionic crystal surfaces.

In the **second calculation**, the Pauli repulsion potential was evaluated as an overlap of the electron densities of tip and sample, and we used a tip charge distribution obtained from DFT calculations rather than using a quadrupole model to calculate the electrostatic force. This calculation is roughly equivalent to the method developed by Ellner et al. when using an exponent of $\alpha =1.0$ for the density overlap [see Eq. (1) in Ref. ^18^].

The input relaxed atomic structure of the $\hbox {CaF}_{2}$ surface, electron density and electrostatic potential were obtained using VASP^72^ with default projector augmented wave (PAW)^73^ pseudopotentials for Ca, F, C and O with a cutoff set to $400\hbox { eV}$. Bloch wavefunctions of this 2 $\times$ 2 unit cell with 2 layers containing 24 atoms were sampled using only the gamma-point, which we consider sufficient for ionic crystals. In both cases the respective potentials and forcefields were evaluated on a regular rectangular grid using the fast Fourier transform based approach described in Ref. ^71^. In both cases, the final forcefield stored on the rectangular grid was further used by the same probe-particle relaxation procedure with the same parameters (lateral stiffness of CO was set to $0.5\hbox { N}/\hbox {m}$). Note that in Ref. ^21^ a lateral stiffness of $0.24\hbox { N}/\hbox {m}$ has been determined for the CO tip. However, as stated by Neu and co-workers^20^, the stiffness of the CO molecule depends on the underlying tip apex and we found better agreement between experiment and theory for the stiffness set to $0.5\hbox { N}/\hbox {m}$. The resulting force was converted to frequency shift using the matrix method with a peak-to-peak amplitude of 1 Å^74^.

### Electrostatic point charge model

While we used two methods to calculate the electrostatic tip-sample interaction in the probe particle model (see above), we additionally calculated the electrostatic interaction between the CO tip and the $\hbox {CaF}_{2}$(111) surface based on the point charge model that is described in detail in Ref. ^39^. In the calculation, the surface ions are represented by single point charges $q_{\text{Ca}} = +1.730\,e$ and $q_{\text{F}} = -0.865\,e$, with the elementary charge *e*^75^. The electrostatic potential and subsequently the electric field are calculated in a 3D grid with a spacing of $5\hbox { pm}$ and a total volume of $1\,\hbox {nm}\,\times \,1\,\hbox {nm}\,\times \,1\hbox { nm}$, centered on the crystal surface. Afterwards, the interaction force between tip and sample is calculated as the force acting on a single point charge *q* in the electric field, and then the frequency shift signal is obtained as described in Ref. ^74^.

As the electric field decays exponentially outside an ionic crystal, the interaction force and the frequency shift will show the same dependence. Therefore, the tip-sample distance *z* and the point charge *q* representing the tip apex cannot be independently determined^39^. As a result, one of the two parameters has to be fixed, and we decided to set the tip apex charge to $q = -0.03\,e$ to represent our CO tip, as reported in Ref. ^27^.

Note that while in the probe particle model the tip-sample distance *z* is defined as the vertical distance between the F$^-_{\text{top}}$ and the tip apex oxygen nuclei, it is defined as the vertical distance between the point charge *q* and the F$^-_{\text{top}}$ nucleus in the electrostatic calculation. As the negative charge density is located slightly in front of the oxygen nucleus^12^, we added an offset $z_{\text{off}} = 80\hbox { pm}$ to the tip-sample distance obtained from the electrostatic calculation to align the *z* axis of both models.

## Supplementary information

Supplementary information.

## Data Availability

The datasets generated during and/or analysed during the current study are available from the corresponding author on reasonable request.
