# Supplementary material for: Quantifying the evolution of atomic interaction of a complex surface with a functionalized atomic force microscopy tip
Source: Sci Rep. 2020 Aug 24;10:14104. doi: 10.1038/s41598-020-71077-9 (PMC7445177; doi:10.1038/s41598-020-71077-9)
Supplement: Supplementary file 1 — Supplementary information. [file 41598_2020_71077_MOESM1_ESM.pdf]

Supplementary Information: Quantifying the evolution of atomic interaction of a complex surface with a functionalized atomic force microscopy tip

Alexander Liebig[1,*], Prokop Hapala[2,3], Alfred J. Weymouth[1], and Franz J. Giessibl[1,+]

[1]Institute of Experimental and Applied Physics, University of Regensburg, D-93040 Regensburg, Germany
[2]Department of Applied Physics, Aalto University, Aalto, Finland
[3]Institute of Physics, Czech Academy of Sciences, Cukrovarnická 10, 162 00 Prague 6, Czech Republic
[*]alexander.liebig@ur.de
[+]franz.giessibl@ur.de

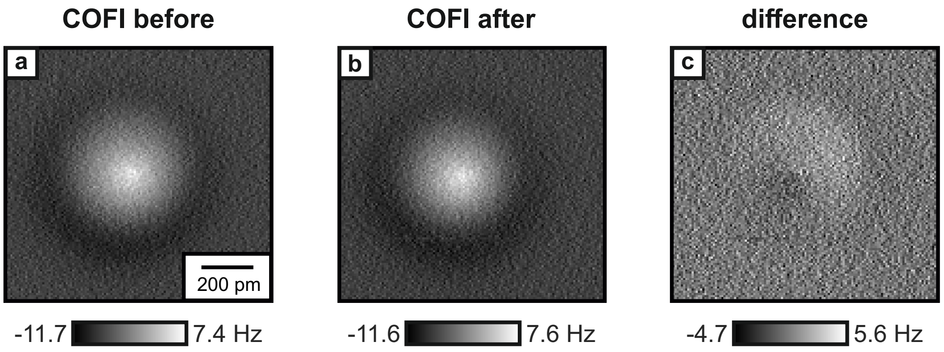

Figure S1. Analysis of COFI images acquired before (a) and after (b) the measurements on $CaF_2(111)$. Both images were measured with zero bias voltage. To compare the two images, they have been centered and subtracted from each other. (c) Difference plot after subtracting (b) from (a). The difference image shows only negligible contrast, proofing that the tip did not change during the experiment. Slight differences can be attributed to a small offset in the imaging height: for the COFI images, the tip-sample distance is set with respect to the STM setpoint [70 pm retracted from the setpoint (−10 mV, −100 pA)]. For low bias voltages, STM images of Cu(111) show an interference pattern of the surface state with adsorbed CO molecules, leading to height differences on the order of 10 pm.

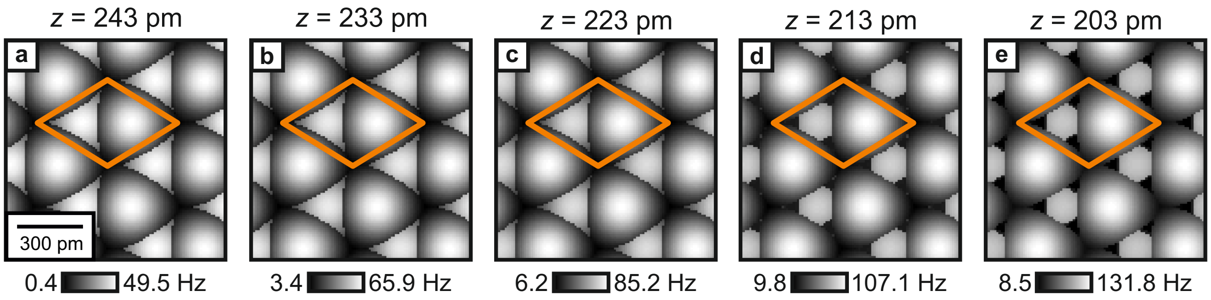

Figure S2. Additional approach images created with the probe particle model at smaller distances as compared to Figure 2 in the main text, illustrating the shrinking of the bright triangular features in the left halves of the unit cells upon approach.

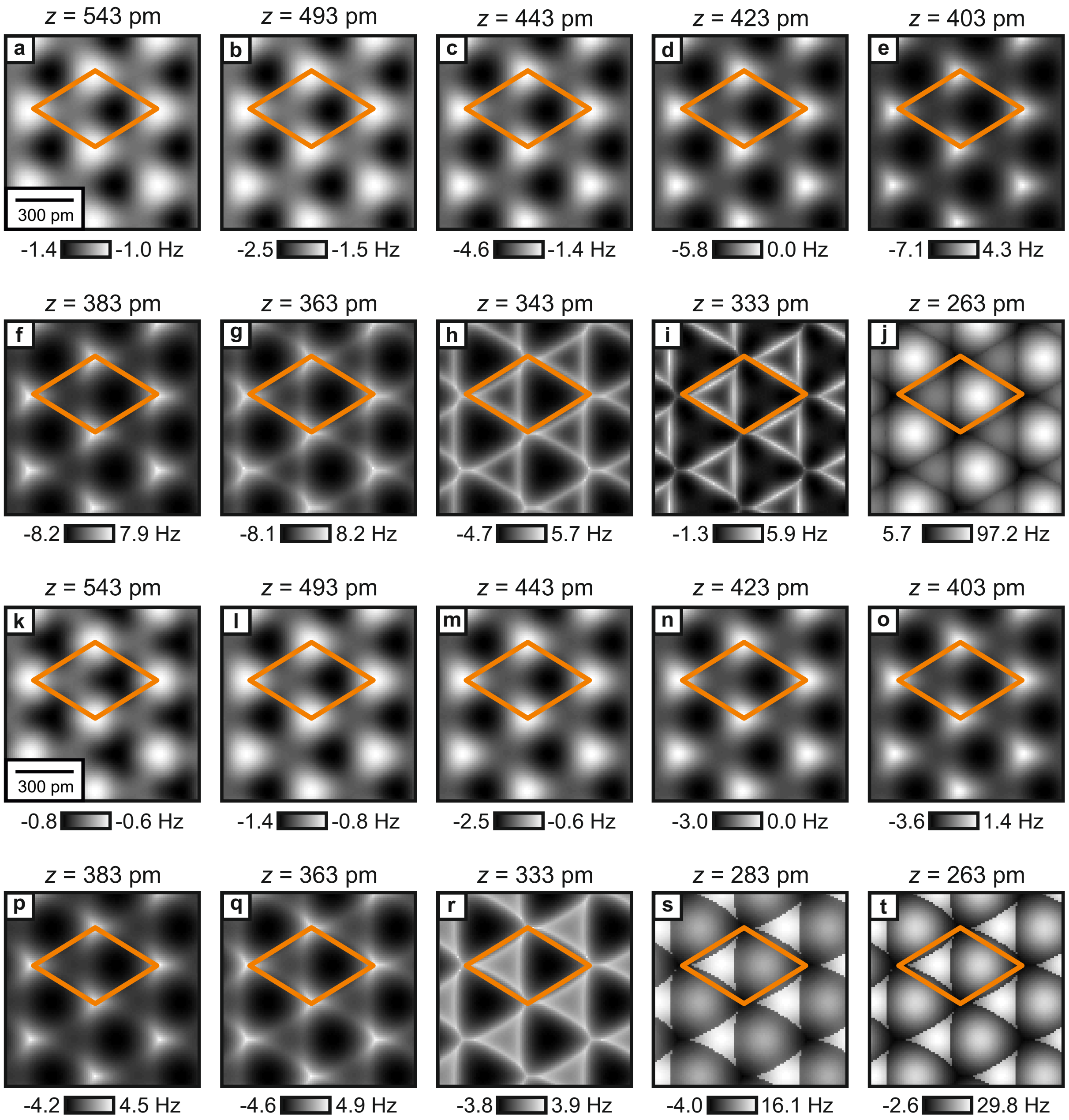

Figure S3. Comparison of constant height images created with the two different versions of the probe particle model. (a)–(j) Images created with the standard model using Lennard-Jones potentials to calculate the contribution of Pauli repulsion. (k)–(t) Images created with the modified density overlap version of the probe particle model.

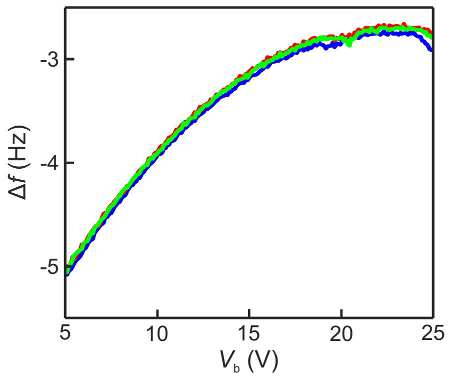

Figure S4. Kelvin parabolas recorded on the three high-symmetry sites of the $CaF_2(111)$ surface. The average apex voltage $V_b = +22.5\,V$ was used as the imaging voltage to compensate the long-range electrostatic tip-sample interaction. The color of the curves follows the scheme of Fig. 2 in the main text.
